# Quickscan assesses risk factors of long-term sickness absence: A cross-sectional (factorial) construct validation study

**DOI:** 10.1371/journal.pone.0210359

**Published:** 2019-01-11

**Authors:** Kaat Goorts, Sofie Vandenbroeck, Tinne Vander Elst, Dorina Rusu, Marc Du Bois, Saskia Decuman, Lode Godderis

**Affiliations:** 1 University of Leuven, Centre for Environment and Health, Leuven, Belgium; 2 Idewe, External Service for Prevention and Protection at Work, Leuven, Belgium; 3 University of Leuven, Faculty of Psychology and Educational Sciences, Leuven, Belgium; 4 Université de Liège, Département des Sciences de la Santé publique, Liège, Belgium; 5 SPMT-ARISTA, External Service for Prevention and Protection at Work, Liège, Belgium; 6 National Institute for Health and Disability Insurance, Department of Benefits, Brussels, Belgium; Medical University Hospital Tuebingen, GERMANY

## Abstract

**Objectives:**

The number of sick-listed employees has increased dramatically worldwide. Therefore, many countries aim to stimulate early and sustainable return to work opportunities to obtain better health outcomes and lower costs for disability pensions. To effectively orientate resources to patients with a high risk of not resuming work spontaneously, it is necessary to screen patients early in their sickness absence process. In this study, we validate “Quickscan”, a new instrument to assess return-to-work needs and to predict risks of long-term sick leave.

**Methods:**

As part of the Quickscan validation process, we tested and compared the reliability and construct validity of the questionnaire in two different populations. First, we conducted a cross-sectional study in which the screening instrument was sent to sick-listed individuals in healthcare insurance. In a second cross-sectional study, sick-listed workers who consulted the occupational health physician for return-to-work assessment were asked to fill out the questionnaire. We compared both samples for descriptive statistics: frequencies, means and standard deviations. Reliability of the scales was calculated using Cronbach’s alpha. Confirmatory factor analysis was performed to evaluate the construct **(factorial)** validity of the studied scales using software package AMOS 24.

**Results:**

The screening tool was shown to be an instrument **with reliable scales** (except for the perfectionism and health perception patient scale) in both populations. The **construct validity** was satisfactory: we found that the hypothesized measurement models with the theoretical factors fitted the data well in both populations. In the first sample, the model improved for scales concerning stressful life events and showed worse fit for person-related factors. Work-related factors and functioning factors both showed similar fit indices across samples. We found small differences in **descriptive statistics**, which we could explain by the differences in characteristics of both populations.

**Conclusions:**

We can conclude that the instrument has considerable potential to function as a screening tool for disability management and follow-up of sick-leave, provided that some adaptations and validation tests are executed.

## Introduction

Long-term sickness absence constitutes a major part of the already substantial costs of sickness absence in Europe [1],[2]. Long-term sickness absence, here defined as absence from work longer than six months due to sickness, is increasing in the 27 European member states and Norway. The Organization for Economic Co-operation and Development (OECD) estimates the costs of disability and sickness to be 2.5 times higher than those of unemployment [1].

According to Kausto et al, many studies have shown that long-term sickness absence is a prognostic marker of future absence from work, early retirement due to ill health problems and mortality [3]. Long-term sickness absence can also be associated with financial difficulties, psychological problems, future unemployment and social exclusion in companies and society [3]. These adverse effects, as well as reduced social contacts with supervisor, colleagues and co-workers, often result in return-to-work barriers that increase the longer the sick leave lasts. All these factors can lead to a downward spiral of deteriorating health and increasing difficulties regarding return to work. Therefore, prevention should be an important component of occupational health policy, not only to reduce sickness-absence-related costs, but also to improve employees’ health, recovery and eventually avoid work disability [2].

A screening method to detect high risk of long-term sickness absence among the large group of sick employees might thus be a useful contribution to support physicians to assess return-to-work needs and assign appropriate support to those with a higher risk for long-term disability. Resources (e.g. financial, human, services, etc.) can thus be provided more efficiently, and the support to the return-to-work process of employees can start earlier.

The development of an evidence-based model to predict long-term sickness absence is a logical and important step in establishing or improving preventive strategies. Most of the current sickness absence models focus almost exclusively on health. Few models include other factors–often work-related or factors addressing social protection. In a predictive model for long-term sickness absence, it is important to realize that cultural and legal differences may have a strong mediating effect on the reported associations with long-term sickness absence [2]. In addition, the impact of many factors (in or outside work) on long-term sickness absence does not necessarily follow a direct path from health deterioration to sickness absence. This means that not only health and work-related factors, but also personal, contextual and other non-work related factors can be relevant in explaining these differences [2].

The idea that a wide range of factors, including work-related psychosocial factors, intrapersonal factors and home/work interference, lead to sickness absence was supported by the Dutch qualitative study Joosen et al. [4]. Workers with common mental disorders also stated that return to work (RTW) was affected by multiple factors related to the work environment and work content, relationships at work and in one’s private life and professional guidance.

Cancelliere et al. [5] even stated that most of the RTW literature has incorrectly focussed on musculoskeletal disorders (e.g., low back pain), although the specific disease-related or biomedical determinants are not the main drivers of patient-centred outcomes such as quality of life and employment. Moreover, identifying modifiable factors is important because certain socio-demographic (e.g., blue-collar work, older age) and some disease-related factors (e.g., presence of rheumatoid factor) fail to provide information on modifiable targets for RTW interventions, even though they are important to identify those at risk for prolonged work disability.

To date, many preventive actions addressing sickness absence have focused on health protection and disease promotion by addressing merely health and work-related factors [2].

Identifying factors causally associated with the increased risk of long-term sickness absence is therefore an important starting point in developing targeted interventions.

Consequently, there is a need for a comprehensive and generic questionnaire that is evidence-based and that enables health care professionals to identify employees at risk for long-term sickness absence.

Several literature reviews aimed to identify factors that predict long-term sickness absence [6], [7], [8]. For example, Beemsterboer et al. reviewed determinants of sick leave frequency and duration. The predicting factors of sick leave duration included gender, age, level of education, marital status, number of children and private life strains, sickness record, perceived health, mental and psychosomatic complaints, physical limitations, lifestyle and the way employers deal with sick employees. Predicting factors were grouped in three categories work characteristics, health characteristics and individual characteristics [6].

A very recent study in Finland examined the association between length of sickness absence and sustainable return-to-work in four important diagnostic categories (depression, anxiety disorders, intervertebral disc disorders and back pain) among public sector employees in Finland. [3]. To predict sustainable RTW (return to work), job characteristics, lifestyle variables and demographic characteristics were analysed. The authors emphasize that RTW not only depends on the type of disease, or on individual or health-related factors but also on the patient’s private and living conditions, features of the work environment as well as cultural and organizational factors, which were not included in their study [3].

To our knowledge, only one psychometric study based a prediction model for long-term sickness absence on existing occupational health survey variables. Roelen et al. [9] created a 15-predictor model, which was reduced to a 9-predictor model, including age, gender, education, self-rated health, mental health, prior long-term sickness absence (LTSA), work ability, emotional job demands and recognition by the management. They observed significant (but moderate) discrimination in the 9-predictor model, but agreed that the instrument is not useful in practice. Apparently, occupational health survey variables do apparently not provide *sufficient* information to adequately discriminate employees at high risk of LTSA from those at low risk. As a consequence, the authors recommend a search for additional predictor variables to improve the discriminative ability of the LTSA prediction model [9]. Since our instrument contains additional predictor variables (such as stressful life events, social support by colleagues, recovery expectations, perfectionism …), we think it might have potential to be more useful in practice.

Therefore, we here aimed to identify factors predicting long-term sickness absence in an extensive literature research. For each identified predictor of long-term sickness absence, we selected items from existing valid and reliable questionnaires [10]. Our Quickscan questionnaire, consisting of very diverse scales, should allow physicians to distinguish between patients with a high and a low risk of long-term sickness absence.

In the current study, we will specifically evaluate the factorial validity (i.e. a type of construct validity) and test for reliability of the scales in two different populations: i.e. patients who consult their occupational health physician with a question concerning return-to-work and health-insured people who enter their 7^th^ week of sickness absence.). We hypothesize that the items of every scale load significantly (p <.001) and in the expected direction on the same dimension, and that the scales show an adequate internal consistency in every sample (Cronbach’s α >.70).

We expect small differences in the average scores on the Quickscan scales, because of the differences in both populations. One population consists of people who are trying to reintegrate in their *current* job (since they consult their occupational health physician), while the other population includes both people who are trying to reintegrate in their current job *and* people who will not be able to return to their previous occupation. The last populations will therefore be more diverse.

## Methods

This study took place in Belgium, where both physicians of healthcare insurance organizations and occupational health physicians have an important role in the return-to-work process. The task of an occupational health physician is to guide reintegration processes within companies (adapted workspace, restricted work, etc.) in cooperation with the healthcare insurance physician. Physicians working for healthcare insurance organizations follow up all patients to assess disability within the first two months of work incapacity and evaluate their rights to sickness benefits. In addition, they help and advice patients to reintegrate after a period of sick leave [11].

Consequently, the instrument was validated in two different study population groups who might benefit from the use of the screening tool. The first population group consisted of all patients who had been on sick leave for six weeks. The second group included employees who consulted their occupational health physician concerning return to work. Since both groups had different characteristics, two analyses were performed. We compared the descriptive statistics, i.e. frequencies, means and standard deviations, between both samples. Reliability of the scales was calculated using Cronbach’s alpha. Confirmatory factor analysis was applied to evaluate the construct validity of the studied scales.

### Variables and instruments

The new screening instrument was constructed based on an extensive review of existing literature and existing questionnaires on (long-term) sickness absence. In a previous study [12], both predictors of long-term sickness absence in general and predictors of long-term sickness absence under certain conditions (e.g. patients with cancer, …) or countries (e.g. Norway) were identified. In total, 21 predictors were identified. Next, we selected items from valid and reliable questionnaires measuring these predictors. Items were selected based on the following criteria: (1) Is the item available in one of both languages spoken in Belgium (Dutch/French)? (2) Has the item been validated in another questionnaire? (3) Is the item easy to understand and applicable in all situations (ranging from more general to very concrete questions).

After the selection of the items for each of the latent factors, we decided to group all latent factors in four large categories, based on the well-known ICF model (e.g. work-, stressful life event-, functioning- and person-related factors) to increase readability and accessibility. We used four main categories, in which work-related factors were considered to be part of the ‘environmental factors’ in ICF, our ‘functioning factors’ were considered to correspond with the ‘body function and structure in ICF’, the ‘personal factors’ were the same as those in ICF, and ‘participation in ICF’ was considered related to ‘stressful life events’ in our questionnaire, because participation is described as the participation of the patient in social life [13].

The questions used in the questionnaire were not subject to any copyright protection since we did not use any complete scales or algorithms to calculate scores from other questionnaires subject to copyright protection. Table 1 presents the structure and content of the patients’ questionnaire (“Quickscan”), the source-questionnaires, the number of items measuring each factor, and the way items or scales were scored. In the newly composed Quickscan questionnaire, all questions were scored on a six-point Likert scale ranging from (1) *totally disagree* to (6) *totally agree*, regardless of the original scale range.

**Table 1:** Structure, content, source-questionnaires and Quickscan scoring*.

Work-related factors	Stressful life events	Functioning	Person-related factors
**Autonomy (5 items)** **Absenteeism screening questionnaire** ***e*.*g*. *I can adjust number and heaviness of my of tasks*** **Learning and development opportunities (4 items)-2** **Vragenlijst beleving en beoordeling van de arbeid (VBBA) [The Questionnaire on the Experience and Evaluation of Work (QEEW)]** ***e*.*g*. *personal growth and development*** **Social support management (2 items)** **Vragenlijst arbeidsreïntegratie [reintegration questionnaire]** ***e*.*g*. *My employer understands my situation*** **Social support colleagues (2 items)** **Vragenlijst arbeidsreïntegratie [questionnaire work reintegration]** ***e*.*g*. *I feel appreciated by my colleagues*** **Physical workload (7 items)** **Vragenlijst beleving en beoordeling van de arbeid (VBBA) [The Questionnaire on the Experience and Evaluation of Work (QEEW)]** ***e*.*g*. *need physical power for your job*** **Workload (6 items)** **Vragenlijst arbeidsreïntegratie/ vragenlijst beleving en beoordeling van de arbeid (VBBA) [reintegration questionnaire/ The Questionnaire on the Experience and Evaluation of Work]** ***e*.*g*. *I work under time pressure*** **Terms of employment (1 items)** **Vragenlijst arbeidsreïntegratie [reintegration questionnaire]** **Emotional burden (1 item)** **Vragenlijst beleving en beoordeling van de arbeid [questionnaire on perception and assessment of labor]** **Turnover intention profession (1 item)** **Vragenlijst arbeidsreïntegratie [reintegration questionnaire]** **Job satisfaction (1 item)** **Vragenlijst arbeidsreïntegratie [reintegration questionnaire]** **Work Expectations (1 item)** **ORO-questionnaire(Obstacles to return-to- work questionnaire**	**Stressful life events (8 items)** **Vragenlijst arbeidsre-integratie [reintegration questionnaire]** ***e*.*g*. *it is hard to find energy to work since my social situation is bad***	**Health perception patient (2 items)** **Disability risk questionnaire** ***e*.*g*. *general health*** ** Psychological distress (7 items)** **SPOC-NL(Somatic Pre-Occupation and Coping Questionnaire)/brief illness perception questionnaire** ***e*.*g*. *How concerned are you about your illness*?** **Pain Perception (3 items)** **SF- 36/ALBPSQ-NL(Acute low back pain screening questionnaire-NL)** ***e*.*g*. *pain in past 4 weeks*** ** Work-health-interference perception (1 item)** **Vragenlijst arbeidsreïntegratie [reintegration questionnaire]** ** Return to work needs (1 item)** **Vragenlijst arbeidsreïntegratie [reintegration questionnaire]** ** Return to work expectations (1 item)** **Vragenlijst beleving en beoordeling van de arbeid [questionnaire on perception and assessment of labor]** ** Recovery expectations (1 item)** ** SPOC-NL(Somatic Pre-Occupation and Coping Questionnaire–NL)** ***e*.*g*. *my treatment will be effective for the cure of my disease*.**	**Fear of colleagues expectations (1 item)** **ORO-questionnaire(Obstacles to return to work questionnaire)** **Perfectionism (4 items)** **Vragenlijst arbeidsreïntegratie [reintegration questionnaire]** ***It’s hard to say no to colleagues***

*All questions are scored on a 6-point Likert scale

### Recruitment of respondents

The Quickscan questionnaire was applied in two settings. The first study population included patients who entered their 7^th^ week of sickness absence according to healthcare insurance organisations (sample 1) and the second population consisted of patients consulting occupational health physicians in preparation of a potential return to work (sample 2).

In the **first study population** all healthcare-insured Belgian people who had entered the 7^th^ week of sickness absence for any reason between 21 August and 3 December 2017 were included. To this end, the disability register of healthcare insurance organisations was used. They sent individual invitations (by email or by regular mail) to patients asking them participate in the study. Every patient received a personal code to enter a website, giving them access to the online questionnaire. This code was linked to their personal file at the healthcare insurance organisation and only the sickness benefit organization could retrieve the link. Sending the questionnaires was part of the legal tasks of the healthcare insurance organisations. The encoded data were transferred via the server of the intermutualistic agency (IMA), a coordination organ of all healthcare insurance organisations. Researchers acquired secure access to the anonymized database by login with Electronic Identity (eID).

Civil servants and self-employed workers were excluded as they have different social insurance systems.

Cross-sectional data from a **second sample** of sick-listed employees were gathered in cooperation with thirty-five voluntary occupational health physicians from eight Belgian external occupational health and safety services and one internal service for occupational health and safety at work. Data were collected from June to October 2017. Occupational physicians were recruited via Co-Prev, the association representing all occupational health and safety (OHS) services in Belgium, and were asked to invite sick-listed employees to participate in the survey.

The inclusion criteria for the patients were: (1) being on sick leave, (2) consulting the occupational physician to discuss reintegration, and (3) French- or Dutch-speaking. Exclusion criteria were: (1) Did not receive sickness benefits (2) Consulted the occupational health physician for any other reason than to discuss reintegration (e.g. periodical medical examination), (3) did not speak French or Dutch (the language of the questionnaire).

For the second study sample, occupational physicians were asked to include every patient consulting to discuss the return-to-work process, for an inclusion period of three weeks. To increase the response rate, individual reminders were sent twice to the occupational health physicians after a period of three weeks. Printed versions of the Quickscan and the informed consent form were delivered to the occupational health physician via regular mail. After three weeks, the occupational health physician was asked to return all questionnaires and informed consent forms. Final ethical approval was obtained from the Ethics Committee of the University Hospitals KU Leuven (S60458).

### Statistical analysis

Analyses were performed with SPSS 24 and AMOS 24. Preliminary data screening on multicollinearity (bivariate correlations between the observed variables higher than *r* = .85 or VIF = 4) and non-normality (skewness index greater than 3; kurtosis index higher than 10) showed no evidence for potential problems for their use in the subsequent analysis in any of the samples [14].

We compared the outcomes of three series of analyses for the two study samples.

First, we compared the **descriptive statistics**, more precisely, frequencies, means and standard deviations, between both samples using paired samples t-tests.

Second, we compared the **reliability** of the study scales, which was evaluated based on Cronbach’s alpha coefficients. The generally agreed-upon criteria for scale reliability is its cut-off value .7 [15].

Third, we compared the **construct (factorial) validity**, which was evaluated through a series of confirmatory factor analyses. Because of the complexity of the study model and the large number of study scales, we tested a measurement model for each category of factors (i.e. work-related factors, stressful life vents, functioning factors, and person-related factors). The model fit of these hypothesized measurement models was evaluated using the following fit indices: the Comparative Fit Index (CFI), the Non-Normed Fit Index (NNFI), the Root Mean Square Error of Approximation (RMSEA), and the Standardized Root Mean Residual (SRMR) [15].

The model fit is considered excellent when CFI and NNFI equal or exceed .95, while good fit is indicated by values above .90. RSEA values below .05 and SRMR values below .09 indicate excellent fit, while values less than or equal to .08 and .10, respectively, indicate good fit [14].

Missing values in the data of the first sample were handled using list-wise deletion. In the second sample, mean substitution was applied. The second sample was rather small, which would have created considerable data loss if list-wise deletion had been used.

## Results

In the first sample, e-mail invitations were sent by all six healthcare insurance organisations to patients who met the inclusion criteria (*N* = 18.736). Of these patients, 3540 respondents fully completed the questionnaire (response rate: 19%). Most respondents were Dutch-speaking (78.2%); with only 37.7% French-speaking.

In the second sample, 276 respondents met the inclusion criteria and were willing to participate in the survey. About 65% were women. The mean age of the respondents was 44.8 years (*SD* = 10.5). Most respondents were Dutch-speaking (86.2%); the French speaking part of Belgium was not equally represented (13.8%). We calculated the duration of the sickness absence until the visit to the occupational health physician. To this end, we used 15^th^ of August as the time of consultation (since this is the middle of our inclusion period), and we used the date the patient filled out as the starting date of his or her disability period). We found that the mean duration of the sick leave before the consultation with the occupational health physician in this study was 42.5 weeks. However, the distribution was skewed. Therefore, we also calculated the median (26 weeks), and the mode (16 weeks).

### Descriptive statistics

Table 2 provides an overview of the means, standard deviations, reliabilities (Cronbach’s alpha coefficients), and factor loadings for the different scales, both for the first and the second sample. The expected relation with long-term sickness absence based on literature and common sense is shown using ‘+’ for a positive relation with a high risk of long-term sick leave, and ‘-‘ for a negative relation with a high risk of long-term sick leave.

**Table 2:** Descriptive statistics (means, standard deviations) and results for reliability tests (Cronbach’s alpha) and confirmatory factor analysis for the different Quickscan scales for sample 1 (n = 3540) and sample 2 (n = 276).

Latent factor: function-related	Cronbach’s α^a^	Mean ^a^	SD^a^	Item	Factor loading^a^
Psychological Distress (+)	.92/.88	2.41/2.52	1.32/1.28	PD1	.65/.62
				PD2	.77/.76
				PD3	.78/.69
				PD4	.78/.63
				PD5	.85/.75
				PD6	.81/.73
				PD7	.86/.80
Health Perception Patient (-)	.79/.53	3.24/2.37***	1.02/1.11	HPP1	.79/.62
				HPP2	.83/.62
Pain Perception (+)	.72/.72	2.30/2.70*	1.21/1.31	PP1	.73/.80
				PP2	.74/.63
				PP3	.60/.65
Work-health interference (+)	/	2.75/3.13	1.66/1.75	/	/
Return-to-work needs (+)	/	1.51/2.48***	1.47/1.63	/	/
Return-to-work expectations (-)	/	2.22/1.65**	1.88/1.70	/	/
Recovery expectations (-)	/	2.75/2.81	1.64/1.56	/	/
**Latent factor: Person-related factors**				Item	Factor loading
Perfectionism (+)	.62/.52	3.71/3.36***	.89/.95	PF1	.43/.36
				PF2	.66/.58
				PF3	.55/.37
				PF4	.55/.54
Fear of colleagues expectations (+)	/	2.30/2.21	1.87/1.88	/	/
**Latent factor: Stressful life-events**				Item	Factor loading
Stressful life events (+)	.87/.83	2.15/1.98***	1.22/1.20	SLI1	.61/.64
				SLI2	.51/.56
				SLI3	.62/.69
				SLI4	.84/.65
				SLI5	.91/.75
				SLI6	.70/.43
				SLI7	.50/.49
				SLI8	.64/.68
**Latent factor: Work-related factor**					
Job satisfaction (-)	/	3.19/2.60**	1.48/1.70	/	/
Terms of employment (-)	/	3.29/2.81	1.26/1.61	/	/
Emotional burden (-)	/	2.40/2.15*	1.86/1.79	/	/
Physical workload (+)	.90/.90	2.65/2.59	1.28/1.40	PWL1	.84/.85
				PWL2	.74/.74
				PWL3	.71/.62
				PWL4	.67/.69
				PWL5	.83/.81
				PWL6	.74/.79
				PWL7	.78/.70
Autonomy (-)	.84/.76	2.02/2.04	1.26/1.21	AU1	.55/.57
				AU2	.76/.67
				AU3	.83/.63
				AU4	.77/.74
				AU5	.68/.60
Social Support by colleagues (-)	.91/.84	3.28/2.59***	1.41/1.56	SSC1	.91/.81
				SSC2	.91/.90
Social Support by Management (-)	.88/.84	3.27/2.63**	1.16/1.68	SSM1	.85/.81
				SSM2	.92/.90
Learning and development opportunities (-)	.81/.83	2.62/2.49	1.41/1.35	LD1	.60/.64
				LD2	.72/.81
				LD3	.86/.81
				LD4	.79/.71
Turnover intention profession (-)	/	3.29/2.71	1.26/2.05	/	/
Workload (+)	.89/.80	2.60/2.68	1.18/1.04	WL1	.71/.62
Work expectations (+)	/	1.98/1.48*	1.20/1.68	WL2	.50/.43
				WL3	.79/.57
				WL4	.88/.77
				WL5	.65/.49
				WL6	.83/.72
				WL7	.77/.65

JS = Job Satisfaction, TOE = Terms of employment, EB = Emotional burden, PWL = physical workload, AU = autonomy, SSC = social support by colleagues, LD = learning and development opportunities, SSM = social support by management, TIP = turnover intention profession, WL = Workload, WE = Work Expectations, SLI = Stressful life events, PD = Psychological Distress, HPP = Health perception patient, PP = Pain perception, RTWE = return to work expectations, WHI = work-health interference, RTWN = Return-to-work needs, RE = Recovery expectations, FCE = Fear of colleagues expectations, PF = Perfectionism, WE = work expectations.

^a^Sample 2/Sample 1 (+) = positive expected relation with high risk of long-term sick leave (-) = negative expected relation with high risk of long-term sick leave

*P <.05;

**P <.01;

***P <.001

Some 1-item scales were used to reduce the length of the questionnaire. The 1-item scales in the questionnaire described in Table 2 are Job satisfaction, terms of employment, return to work needs, turnover intention profession, Work-health interference perception, emotional burden, fear of colleagues’ expectancies, work expectations, return to work expectations and recovery expectations. Some other scales need more items because the scale has more dimensions (e.g. stressful life events).

Regarding work-related factors, the first sample demonstrated significantly lower mean scores on job satisfaction (p = .002), emotional burden (p = .052), social support by colleagues (p <.000) and social support by management (p = .006). Since we expect these factors to be negatively related to risk of long-term sickness absence [5], a lower score in the first sample would suggest that there is a higher risk of long-term sick leave among patients in the insurance medicine settings (sample 1). However, the lower average score on work expectations (p = .052) in the first sample might reflect a lower risk of long-term sickness absence, since this factor is supposed to be positively related to a high risk of long-term sick leave [16]. Equal mean scores were found in terms of employment, physical workload, autonomy, learning and development opportunities and workload.

The mean score for stressful life events was significantly (p = .001) lower in the first sample. Since we expect stressful life events to positively relate to long-term sickness absence [5], this means that a lower risk of long-term sick leave can be expected. Concerning the person-related factors, the first sample had significantly (p <.001) lower mean scores for perfectionism (p = .001), and equal mean scores for fear of colleagues’ expectations. Therefore, we can expect a lower risk of long-term sickness absence based on this factor.

For the functioning factors, the mean scores for health perception patient (p <.001) and return-to-work expectations (p = .003) were lower in the first sample. Both of these factors are expected to be negatively related to long-term sickness absence [17], [18]. Therefore, we expect a higher risk of long-term sickness absence in the first sample. Return-to-work needs (p <.001) and pain perception (p = .015) demonstrated significantly higher scores in the first sample. These factors are positively related to a high risk of long-term sickness absence [5]. Therefore, a higher risk of long-term sickness absence is expected in the first sample. Psychological distress, recovery expectations and work-health interference showed no difference in mean score across samples.

### Reliability

Most scales were found to be reliable (see Table 2). Psychological distress (.92/.88 for Sample 2 and 1 respectively), pain perception (.72/.72), stressful life events (.87/.83), physical workload (.90/.90), autonomy (.84/.76), social support by colleagues (.91/.84), social support by management (.88/.84), learning and development opportunities (.81/.83), workload (.89/.80). Based on the Cronbach’s alpha, we observed a problematic reliability (α <.70) in the health perception patient scale for the first sample (.79/.53) and for both samples in the perfectionism scale (.62/.52).

### Construct validity

The results of the confirmatory factor analyses are shown in Table 3. All four hypothesized models (one for each category; i.e. work-related factors, stressful life events, functioning factors and person-related factors) fitted the data well in both samples. In the first sample, the model for stressful life events was better (CFI = .96>.90, NNFI = .95>.86, RMSEA = .06 < .14, SRMR = .03>.07) and the model for person-related factors showed a worse fit in comparison with the second sample (CFI = .94 <.97, NNFI = .89 <.93, RMSEA = .07>.06, SRMR = .03 <.04). The measurement model for work-related factors and for functioning factors showed similar fit indices in both samples (CFI, NNFI, RMSEA, SRMR). This means that the models fitted the data well for both samples.

**Table 3:** Main results of confirmatory factor analysis: Latent factors fit indices and a model comparison for sample 1 (n = 3540) and sample 2 (n = 276).

Model	# latent factors	Latent factors	*χ2*	df	CFI	NNFI	RMSEA	SRMR
	**Work-related factors**							
Sample 2	11 factors	JS, TOE, EB, PWL, AU, SSC, SSM, LD, TIP, WL, WE	5440.08***	414	.90	.88	.06	.06
Sample 1	11 factors	JS, TOE, EB, PWL, AU, SSC, SSM, LD, TIP, WL, WE	782.22***	336	.90	.87	.07	.06
	**Stressful life events**							
Sample 2	1 factor	General factor	121.30***	20	.90	.86	.14	.07
Sample 1	1 factor	General factor	313.63	20	.96	.95	.06	.03
	**Functioning-related factors**							
Sample 2	7 factors	PD, HPP, PP, RTWE, WHI, RTWN, RE	257.30***	87	.93	.90	.08	.06
Sample 1	7 factors	PD, HPP, PP, RTWE, WHI, RTWN, RE	1482.35***	87	.93	.90	.07	.05
	**Person-related factors**							
Sample 2	2 factors	FCE, PF	10.08**	5	.97	.93	.06	.04
Sample 1	2 factors	FCE, PF	87.38***	5	.94	.89	.07	.03

JS = Job Satisfaction, TOE = Terms of employment EB = Emotional burden, PWL = physical workload, AU = autonomy, SSC = social support by colleagues, LD = learning and development opportunities, SSM = social support by management, TIP = turnover intention profession, WL = Workload, SLI = Stressful life events, PD = Psychological Distress, HPP = Health perception patient, PP = Pain Perception, RTWE = return to work expectations, WHI = work-health interference, RTWN = Return to work needs, RE = Recovery expectations, FCE = Fear of colleagues expectations, PF = Perfectionism, WE = Work Expectation

*P <.05;

**P <.01;

***P <.001

Based on the factor loadings from the confirmatory factor analysis (see Table 2), we found one problematic factor loading (<.40) in the perfectionism scale. In sample 2, this loading was nearly problematic (.43); in sample 1, it decreased to a problematic level (.36). We did not leave out any of the problematic items, because we wanted to compare the fit indices of the same models in both populations, including the same items.

## Discussion

The aim of the current study was to validate the Quickscan (construct/factorial validity), a questionnaire assessing predictors of long-term sickness absence, across two independent Belgian populations. We found reliable scales and valid constructs in both samples. As we expected, we found different results for both groups, because of their different population characteristics.

This study showed that the screening tool is an instrument **with reliable scales** (except for the perfectionism and health perception patient scale) in both populations. A possible pathway to improving the unambiguous understanding and increasing the reliability is a reformulation of these two variables. Therefore, we will perform a qualitative research with patients to test for content validity. The **construct validity** was satisfactory: we found that the hypothesized measurement models with the theoretical factors fitted the data well in both populations. The model for stressful life events fitted the data better in the insurance medicine population (sample 1) than in occupational medicine (sample 2). Model fit for the measurement model with the person-related factors was worse in insurance medicine (sample 2). The reason for these differences is not clear. Similar fit indices were found in sample 1 and 2 for the models concerning work-related factors and functioning factors.

We found slightly different results in both populations. In general, the patients in the insurance medicine (sample 1) seem to have a higher risk of long-term sickness absence based on the functioning factors and work-related factors, and a lower risk based on stressful life events and person-related factors. This difference in risk factors might be explained by Brouwer et. al, who argue that the Phase Model of Occupational Disability stresses the phase specificity of risk factors: Physical and injury factors (similar to our work-related and functioning-related factors) are determining predictors of occupational disability in the acute phase (up to 1 month), whereas psychosocial factors (similar to our stressful life events and person related factors) have stronger predictive value in the subacute (2–3 months) and chronic phases of occupational disability (more than 3 months) [19]. Hence, the patients sampled in occupational medicine had a median duration of work disability of 26 weeks in this study, before they consulted the occupational health physician. Most of them were in the subacute or the chronic phase, while the other patient population had a sickness absence duration of six weeks, which reflects the period just passed the acute phase. Therefore, physical and injury factors, which are more predictive in the acute phase, might predict long-term sickness absence better for the group of patients in insurance medicine (sampled after six weeks of sick leave), and the psychosocial factors might predict long-term sickness absence better in the group of patients sampled in occupational medicine after a median time of 26 weeks (subacute/chronic phase). This means that all factors included in the questionnaire are to some extent important in both settings. Nevertheless, we observed differences in risk factors, which can be explained by Brouwer et al. (2011). Therefore, in the further validation of the instrument, adjusted weights assigned to the questions in different contexts will be needed in order to correctly assign sick-listed workers according to their risk of long-term sickness absence. The algorithm that will predict the time until return to work will be adapted to the context in which the questionnaire is applied. This is subject of a current study set up to assess the predictive validity [20].

One of the major strengths of this research is the comparison of all results over two population samples in two different contexts. Hence, the instrument might be useful in both contexts because of the important role for both physicians in return to work. However, since the patients have slightly different characteristics, it is interesting to compare the results of both patient samples. Another strength of this research are the large samples, which made the analysis more reliable.

Some limitations to this study are worth mentioning. First, cross-sectional data were used in this study. Since we did not follow the patients over a longer period of time and we did not have an objective measure of sickness absence, we were not able to evaluate the predictive validity of the Quickscan questionnaire. Hence, a fruitful path for further research is to investigate whether the Quickscan scales could predict time until return to work, thereby evaluating the predictive validity of the instrument. This kind of predictive validity study has already been set up and will be executed when data are available [10]. Second, we measured predictors of sickness absence in two populations, at a different moment in time. For the first group, we measured the risk of long-term sickness absence after six weeks. For the second group, data collection was spread over a longer period of time and generally much later after the start of the sickness absence (since it was at the time of the consultation with the occupational health physician). This makes it harder to compare the results between both populations. However, it should be mentioned that this would be the reality when implementing the questionnaire. If the questionnaire were used in occupational medicine, it would not be used at one fixed time point.

Another limitation might be that we did not test the model as a whole. However, the four selected constructs based on the ICF (i.e. work-related, person-related, stressful life-event-related and function-related) make the questionnaire easier to interpret, but are not the purpose of any kind of analysis. Therefore, performing a second order model test (including the four categories) would be very complex, with a low model fit. Hence, we expect cross loadings within one category because factors might correlate. The four constructs will never be used for scoring purposes. However, the latent variables will have a score (for example, a weighted score for health perception),.

In future studies, we will define cut-off values for each factor (21 factors in the questionnaire). This way, we will be able to generate an automatic ‘report’ for both patient and physician about the ‘risk factors’ based on the answers to the questionnaire and with adjusted weights according to the context. As a result, the consultation can focus on the most problematic aspects. If the report shows no or low risk factors, and the physician does not consider the patient as at risk of long-term occupational disability (based on the patient’s file), he/she can prioritize other patients.

For the patients, this would mean that ‘high-risk’ patients, who are reporting severe issues on one or more questionnaire items, can get priority in receiving early follow up and support. Patients who do not report many issues will be considered temporarily as patients who will be able to resume their work independently. This kind of procedure of course entails the risk that people who are providing socially desirable answers to the questionnaire will not receive the support they might need within the first months of their sick leave. The questionnaire will never replace the estimation of the physician, who will always stay in charge. However, the questionnaire can be valuable information in the medical file of the patient, which can be shared with different partners involved in the RTW process.

Overall, the instrument shows considerable potential to function as a screening tool in both settings, provided that some adaptations are executed. After validation and implementation in Belgium, we aim to test and implement the screening tool in other countries with a different social security system, culture and settings to detect the risk of long-term sickness absence in an early phase and thus support both patients and physicians to strive towards early return to work. Further research is necessary to determine the predictive value of the instrument with regard to actual time until return to work. Important is that predictive validity should be tested in both contexts, since we found some important differences in both populations. Furthermore, the content validity needs to be examined in a study using qualitative research with patients.

## Supporting information

S1 Dataset DatasetN = 276.xlsx. (XLSX)

S2 Dataset DatasetN = 3540.xlsx. (XLSX)
